# Free-breathing real-time cardiac cine MR for evaluation of left-ventricular function: Comparison to standard multi-breath-hold cardiac cine MR in 50 patients

**DOI:** 10.1186/1532-429X-18-S1-Q50

**Published:** 2016-01-27

**Authors:** Masashi Nakamura, Tomoyuki Kido, Yoshiaki Komori, Michaela Schmidt, Christoph Forman, Kouki Watanabe, Teruhito Mochizuki

**Affiliations:** 1 grid.255464.40000000110113808Radiology, Ehime University, Toon, Japan; 2 grid.459909.80000000406406159Radiology, Saiseikai Matsuyama Hospital, Matsuyama, Japan; 3 Siemens Japan K.K, Tokyo, Japan; 4 grid.459909.80000000406406159Saiseikai Matsuyama Hospital, Matsuyama, Japan; 5 Siemens Healthcare GmbH, Erlangen, Germany

## Background

Electrocardiogram (ECG)-gated breath-hold cardiac cine magnetic resonance imaging (MRI) is generally accepted as the gold standard for left-ventricular (LV) volume assessment. However, it may fail in patients with arrhythmia, impaired breath-hold capacity, and poor ECG gating. Recently, sparse real-time (RT) cine using a prototype sequence with sparse sampling and iterative reconstruction has been proposed to accelerate cine MRI (Kido et al. SCMR; 2015). The purpose of this study was to evaluate the diagnostic quality and accuracy of sparse free-breathing (FB) RT cine MRI for the quantification of LV function compared with standard multi-breath-hold cine MRI.

## Methods

50 patients underwent both standard segmented cine MRI (Acc. factor 3) and sparse FB RT cine with a prototype sequence using sparse sampling and iterative reconstruction (acc. factor 12.8) on a clinical 3T MRI scanner (MAGNETOM Skyra, Siemens Healthcare, Erlangen, Germany). The cine images were obtained in a stack of 8 short-axis slices spanning the entire LV from base to apex (temporal/spatial resolution: 41 ms/1.7 × 1.7 × 6 mm^3^). The image quality, ejection fraction (EF), end-diastolic volume (EDV), end-systolic volume (ESV), stroke volume (SV), and LV mass for sparse FB RT cine and standard cine were compared.

## Results

All sparse FB RT cine showed acceptable diagnostic image quality. Standard cine and sparse FB RT cine showed good agreement: EF (60.3 ± 10.3% for standard vs. 58.8 ± 10.7% for FB RT; p = 0.09); EDV (132.5 ± 36.7 ml vs. 133.9 ± 33.8 ml; p = 0.51); ESV (54.8 ± 27.4 ml vs. 57.3 ± 27.3 ml; p = 0.09); SV (77.6 ± 15.9 ml vs. 76.6 ± 14.3 ml; p = 0.53); LV mass (87.6 ± 33.7 ml vs. 81.3 ± 31.3 ml; p < 0.001). The intra-observer and inter-observer agreement for all parameters was good.

## Conclusions

Sparse FB RT cine MRI evaluates LV function with good accuracy compared with conventional multi-breath-hold cine MRI. For patients with impaired breath-hold capacity, FB RT cine MRI may be clinically useful for quantitative assessment of LV function.

**Figure 1:**
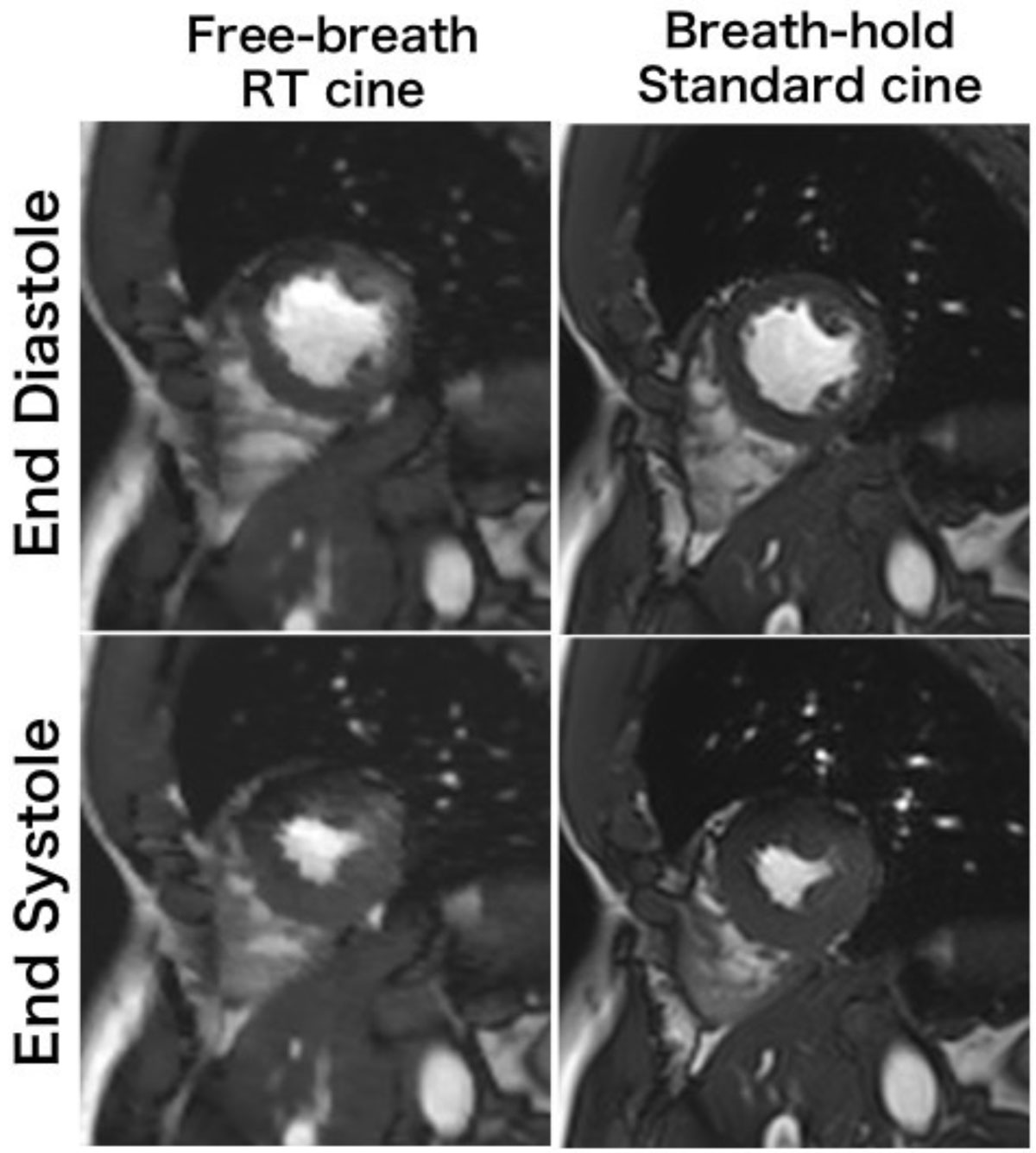
Figure 1

